# Comprehensive Genetic Testing for Clinical Decision‐Making in a Patient With Congenital Hyperinsulinism

**DOI:** 10.1155/genr/7281653

**Published:** 2025-12-12

**Authors:** Matthias Begemann, Johannes Alexander Tobias Boy, Florian Kraft, Hendrik Vossschulte, Winfried Barthlen, Sebastian Kummer, Thorsten Orlikowsky, Adrin Torosoglu, Thomas Eggermann, Ingo Kurth, Miriam Elbracht, Angeliki Pappa

**Affiliations:** ^1^ Center for Human Genetics and Genomic Medicine, Faculty of Medicine, RWTH Aachen University, Aachen, Germany, rwth-aachen.de; ^2^ Department of Pediatrics, Faculty of Medicine, RWTH Aachen University, Aachen, Germany, rwth-aachen.de; ^3^ Pediatric Surgery and Urology, Children’s and Youth Hospital Auf der Bult, Hannover, Germany; ^4^ Department of Pediatric Surgery, Protestant Hospital of Bethel Foundation, University Hospital OWL, Campus Bielefeld Bethel, University of Bielefeld, Bielefeld, Germany, uni-bielefeld.de; ^5^ Department of General Pediatrics, Neonatology and Pediatric Cardiology, Medical Faculty, University Hospital Düsseldorf, Heinrich-Heine-University, Düsseldorf, Germany, uni-duesseldorf.de; ^6^ St. Josef-Stift Hospital Sendenhorst, Sendenhorst, Germany

**Keywords:** comprehensive genetic, congenital hyperinsulinism, long-read sequencing, neonatal hypoglycemia, therapeutic algorithm in CHI

## Abstract

This case study examines a preterm newborn with autosomal recessive *ABCC8* gene‐related diffuse congenital hyperinsulinism (CHI). The interdisciplinary management of the patient, including advanced genetic testing and long‐read sequencing, finally led to the molecular diagnosis. These findings were relevant for the immediate decision on further treatment options highlighting the importance of differentiating between focal and diffuse CHI forms, and for providing the family with counseling on the recurrence risks and prenatal diagnostic options. In summary, this study illustrates the clinical and genetic intricacies of CHI, emphasizing the significance of comprehensive genetic analysis in diagnostics and tailored treatment. The case advocates for the integration of state‐of‐the‐art genetic diagnostic technologies in combination with clinical interdisciplinary management to improve patient outcomes.

## 1. Introduction

The regulation of glucose metabolism is one of the essential vital processes of life and is controlled by a complex interplay of insulin, glucagon, and other hormones. In addition to more common diseases such as Type 1 and Type 2 diabetes, rare congenital disorders of glucose metabolism have been described. These include early childhood congenital hyperinsulinism (CHI), which poses a serious risk to the health and life of the newborn child [1]. Single gene mutations directing the insulin secretory pathway can cause CHI. Even if the transient or acquired forms may be managed within days, neurodevelopmental disturbances as results of severe hypoglycemia have been reported in up to one‐third of the children [1, 2].

CHI is a rare genetic condition in which the pancreas produces inadequately high amounts of insulin, leading to low blood glucose levels (hypoglycemia). CHI is related to pancreatic beta‐cell disorders causing dysregulated insulin secretion. The clinical picture is characterized by repeated hyperinsulinemic hypoglycemia [1–4]. This may lead to increased risk of seizures, impaired global development, and permanent brain damage if not diagnosed and treated timely [1]. To protect the affected children, adequate diagnostic procedures including genetic work‐up and therapy are crucial [4]. The incidence of CHI is approximately 1 : 28,000–1 : 50,000 in Western populations [1, 2, 5] and can be 1 : 2500 in populations with higher rates of consanguinity [1, 2].

Treatment options include medication, dietary modifications, and, if indicated, surgery, but the specific approach depends on the underlying cause of CHI. Diazoxide is recommended as first‐line treatment of hyperinsulinism. If effective, it suppresses the insulin secretion by opening the ATP‐sensitive potassium channels (KATP) of beta‐cells. Regarding second‐line options for patients with CHI who are diazoxide‐unresponsive or have unacceptable diazoxide side effects, somatostatin analogs are recommended. Those include octreotide, long‐acting octreotide, and lanreotide. For patients with genetically proven diffuse CHI, first‐line treatment is medical therapy; however, pancreatectomy might be necessary in cases of uncontrollable hypoglycemia [6].

The most common genetic causes are pathogenic variants in the genes *KCNJ11* and *ABCC8* located on Chromosome 11p15.1, which encode the two subunits, SUR1 (sulfonylurea receptor) and Kir6.2 (potassium pore), of the hetero‐octameric beta‐cell plasma membrane KATP channel, respectively [7, 8]. Inactivating mutations are responsible for the distributed channel regulation. The hereby caused reduced efflux of K^+^ causes inappropriate membrane depolarization and dysregulated Ca^2+^ entry resulting in inadequate insulin secretion.

CHI may manifest with a complex genetic inheritance pattern as variants in *ABCC8* and *KCNJ11* can cause different forms of CHI with autosomal recessive and autosomal dominant inheritance. Moreover, a third, non‐Mendelian inheritance pattern is known that leads to the focal form of CHI and is even more difficult to detect genetically.

In the latter form, the hyperinsulinism is limited to a specific area of the pancreas; it results from two simultaneous changes in the beta cells of the affected region: loss of heterozygosity for the chromosomal Region 11p15 due to a (somatic) loss of the maternal allele [4, 9]. As a result, a paternally inherited germline mutation in *ABCC8* or *KCNJ11* becomes “homozygous” or “hemizygous” due to the absence of the second allele leading to hyperinsulinism in the affected beta‐cells.

## 2. Results

### 2.1. Clinical Presentation

The patient was born preterm (34 + 3 gestational weeks) and large for gestational age (LGA) (weight 3330 g, length 51 cm). She presented with hypotonia and recurrent hypoglycemia, minimum glucose 8 mg/dL, APGAR 6/8/9, was referred to Neonatal Intensive Care Unit (NICU), received intravenous glucose 10% and then continuous glucose infusion. The need for daily total glucose went up to maximum 45 g/kg/d, including 8.7–14 mg/kg/min intravenously (the recommended daily carbohydrate administration at day 5 being 14 g/kg/d).

Glucagon was given for persistent episodes of hypoglycemia. Proof of high insulin levels related with hypoketotic hypoglycemia led to further diagnostic work‐up for CHI. Treatment with diazoxide on Day 8 postpartum and stepwise increase of dosage up to 20 mg/kg/d did not result in clinical stabilization. Treatment was accompanied by hydrochlorothiazide because of edema. This therapy was stopped on postnatal day 28. Treatment with octreotide was initiated with dose up to 40 μg/kg/d and could decrease hypoglycemic episodes. Few hypoglycemias in the next days could be treated with dextrose gel 40% or glucagon injection. On day 44, the total daily carbohydrate intake (oral and intravenous) was 31 mg/kg/d. Parents were instructed to use the continuous glucose monitoring system.

After detailed discussion of the therapeutic options with the parents, the child was transferred to a specialized surgical unit, and partial pancreatectomy was performed. Within the first laparotomy at the 49th day of life, about 30%–40% of the pancreas was resected from the tail. The mapping biopsies from the tail, corpus, and processus uncinatus showed a diffuse form of CHI. Because the intravenous glucose intake could not be reduced satisfactorily, a second laparotomy was performed on the 71st day of life. Another part of the formerly resected tail was cut, and finally 50% of the pancreas was resected. The postoperative course included continuous oral feeding via a jejunal tube and intravenous glucose dispensation. Both could be replaced by gastral bolus dispensations with a composite of formula feeding and maltodextrine. Octreotide dose was continued. The glucose need was 15.6 g/kg/d at discharge after 2 months.

Postoperative course: As blood‐glucose control (fasting tolerance only for 3 h, recurrent hypoglycemia) was still suboptimal; further optimization of octreotide and of nutritional supplementation was intended: Octreotide was given 3×/day with 80 μg (29.6 µg/kg/d) and supplementation of carbohydrates with oatmeal 8 g per feeding.

The intravenous glucose infusion ranged between 14 and 31 mg/kg/min and later even after surgery 40–80 mg/kg/min so octreotide had to be maintained.

The increased insulin action can be demonstrated by increased glucose requirement (e.g., > 8 mg/kg/min).

Glucose levels were manageable under treatment with octreotide. A therapy trial with lanreotide is planned.

### 2.2. Genetic Findings

**Figure 1:**
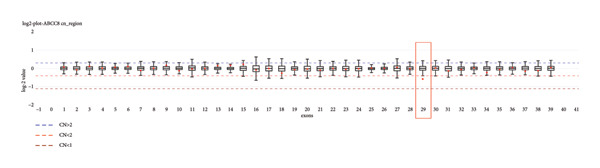
Copy number boxplots generated using the CNVizard tool. The boxplots visualize the bias corrected log2 depth value, calculated using CNVKit over a reference of genome samples. The box is made up out of the 0.25 and 0.75 quartile of log2 values present in the reference. The whiskers show the minimum and maximum values contained in the reference. The dashed lines represent the cutoff values suggested by CNVkit. The red dot highlights the log2 value of the sample analyzed. The red dot is located below the lower whisker of the boxplot generated for Exon 29, therefore suggesting a single‐exon deletion.

**Figure 2:**
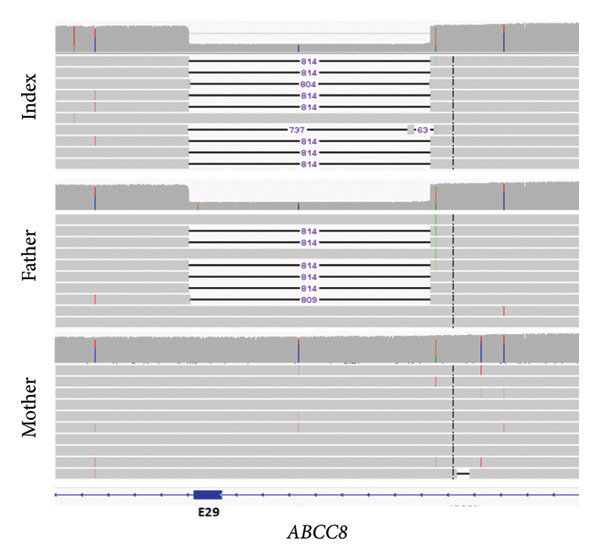
IGV view of the long‐read nanopore data showing the 814 bp deletion affecting exon 29 and intron 28 in the index patient, the father, and the intact allele of the mother (the upper part in each panel represents the coverage of every single base and the lower part shows the individual reads spanning the region).

Because of strong suspicion of CHI, genetic testing was initiated. Trio‐exome sequencing analysis was performed, and the analysis focused on *ABCC8*, *KCNJ11*, and genes associated with clinically overlapping symptoms. The trio‐exome analysis revealed a clear pathogenic nonsense variant in *ABCC8* inherited from the clinically nonaffected mother, resulting in a premature stop codon (*ABCC8*, NM_000352.6:c.2800C>T, depth: 92×, allele distribution 43/49) which strengthened the suspicion of a diffuse autosomal recessive form of familial hyperinsulinism, due to the mostly paternal inheritance of the monoallelic‐recessive focal forms of CHI. Remarkably, no single nucleotide variation (SNV) could be detected on the paternal allele, but the copy number analysis indicated a deletion affecting Exon 29 of the *ABCC8* gene in the patient and the father (Figure 1). To confirm this finding with an independent method, multiplex‐ligation–dependent probe amplification (MLPA) analysis for *ABCC8* was initiated. Surprisingly, the paternal deletion could not be confirmed by MLPA. Nevertheless, the clinical course of the patient was highly suggestive for a diffuse autosomal recessive form of CHI. Therefore, we designed a long‐range PCR, ranging from Exon 28 to Exon 30 of *ABCC8* spanning the potential breakpoints that may cause Exon 29 deletion. The resulting amplicons of the index‐patient and her father were indicative for a larger deletion in this region. To delineate the breakpoints, we applied long‐read sequencing using Oxford Nanopore Technologies (ONT). The results of this long‐read sequencing approach confirmed the presence of an 814 bp deletion (NM_000352.6:c.3558‐727_3647delinsC, p.[Arg1186Serfs∗4], Chr11:17402664_ 17403480delinsG [hg38] Figure 2), affecting almost the entire exon 29 and a large part of intron 28. Interestingly, the MLPA probe for exon 29 is located upstream of the exon in intron 29 with only a small overlap to the coding region of exon 29. Consequently, the deletion did not disrupt the hybridization process of the MLPA and was therefore not detectable by this approach (Figure 3).

Figure 3(a) MLPA result for *ABCC8*. The MLPA did not confirm the deletion of exon 29 in the *ABCC8*‐Gene. (b) Schematic representation of the deletion (dashed area) and the localization of the MLPA probe for exon 29.

**Figure:**
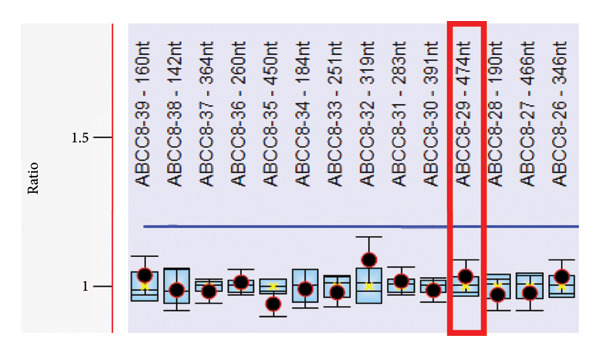
(a)

**Figure:**
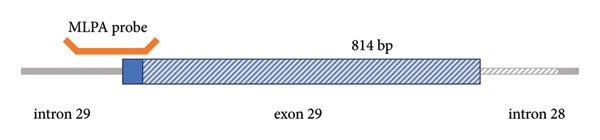
(b)

Based on compound heterozygous pathogenic variants in *ABCC8*, (NM_000352.6:c.2800C>T (Clinvar Variation ID: 1705703) and NM_000352.6:c.3558‐727_3647delinsC), the diagnosis of an autosomal recessive CHI could be confirmed in the patient.

## 3. Discussion

CHI is associated with a high risk of seizures, developmental delay, and brain damage if it is not recognized quickly and treated appropriately. To avoid lifelong consequences, a rapid and reliable diagnosis is therefore necessary to be able to take the right treatment measures. For clinical treatment, it is crucial to differentiate between the focal and the diffuse form of CHI. The aim of treatment for hyperinsulinism is to stabilize plasma glucose concentrations within the normal range of 70–100 mg/dL. Oral and intravenous glucose and glucagon administration might need medical treatment comprising diazoxide and as second‐line treatment somatostatin analogs [6]. The focal form is preferentially treated by surgical intervention, an approach that is only of limited use in the diffuse form. For the diffuse forms, carbohydrate administration, medical treatment as mentioned above and thereby maintaining glucose levels, is paramount.

In this case, the clinical course and the detection of a known pathogenic variant (on the maternal allele) underlined the necessity of a further genetic workup including nonroutine diagnostic tools.

Several factors finally led to the diagnosis in this case: the clinical course of the patient, the initial detection of a pathogenic variant on the maternal allele, and the indication for a possible deletion on the paternal allele via exome sequencing.

The genetic diagnosis might guide further diagnostic workup: In case of the paternally inherited heterozygous mutation in *ABCC8* or *KCNJ11* imaging with F‐DOPA/PET CT (F‐fluorodihydroxyphenylalanine positron emission tomography/computed tomography) should be performed for the potential localization of the focal form. Surgical treatment should be considered for the proven focal forms or for severe cases resistant to therapy.

By verifying the diagnosis of CHI and the heterozygous carrier status of both parents for pathogenic variants in *ABCC8*, the recurrence risk for further children with CHI is 25% and should be discussed in genetic counseling. Based on the obtained molecular results, prenatal testing can be offered to the couple, keeping in mind the necessity of using nonroutine genetic methods.

The presented case underlines the importance of state‐of‐the‐art laboratory methods, such as long‐read sequencing for diagnostics, and the potential of this method to detect genetic alterations that might escape assays like MLPA due to their inherent limitations, such as the limited target regions. Additionally, algorithms and tools that can detect smaller deletions affecting one or more exons of a gene are of great importance in the diagnostic workup.

Our findings underline the importance of genetic testing not only to establish a diagnosis in CHI but also for informing the prognosis and guiding individualized treatment strategies based on genotype–phenotype correlations. In addition to its diagnostic value, our study highlights the prognostic relevance of genetic testing in CHI, particularly emphasizing the advantages of nanopore long‐read sequencing, which enables comprehensive detection of structural variants and phasing of compound heterozygous mutations—capabilities that surpass those of isolated, gene‐specific testing approaches.

## 4. Methods

### 4.1. Consent

Written informed consent for clinical testing and publication was obtained from the parents.

### 4.2. Exome Sequencing

Exome sequencing was performed with DNA from peripheral blood of the patients and their parents. Library preparation and enrichment was done using IDT xGen DNA Library Prep, EZ UNI kit/xGen Hybridization and Wash Kit together with xGen Exome Hyb Panel v2. The libraries were sequenced on a NovaSeq6000 platforms (Illumina) with 2 × 159 cycles, average exome coverage was 153×, with 97.5% > 30×. Mean coverage at *ABCC8* was 184×, with 100% of the gene covered above 30×.

Alignment (genome build GRCh38), base calling, and secondary analysis were conducted using BCL convert (v4.2.4) and Illumina DRAGEN Germline pipeline (v4.1), respectively. Annotation and bioinformatic prioritization of variants were carried out using KGGSeq (v1.1, 07/Feb./2019). Variants with a minor allele frequency > 0.75% in public databases (i.e., gnomAD) and synonymous variants were not considered. CNVkit (v.0.9.10) [10] and CNVizard (v.0.3.1) [11] were used for CNV calling.

### 4.3. MLPA

MLPA analysis was performed according to the manufacturers’ protocol using SALSA MLPA Probemix P117 ABCC8 Version C3 (MRC Holland, Amsterdam, Netherlands).

### 4.4. Long‐Read Sequencing Using Nanopore Technology

The *ABCC8* Exons 28–30 were amplified by long‐rage (LR)‐PCRs (Primer Sequences ABCC8‐E29‐BP.1‐LRF, ctc​gtc​tcc​ctc​agc​aca​tc; ABCC8‐E29‐BP.1‐LRR; ttg​gca​gct​gtg​agg​aag​ag; ABCC8‐E29‐BP.2‐LRF, ctc​cct​cag​cac​atc​cca​tc, ABCC8‐E29‐BP.2‐LRR, ggc​cag​ggt​aga​ggg​gaa​ta). LR‐PCR was carried out with the AccuPrime Taq DNA Polymerase (Thermo Fisher Scientific, Waltham, Massachusetts, USA). The PCR product was cleaned up with 0.4× CleanNA CleanNGS beads (CleanNA, Waddinxveen, Netherlands). Nanopore sequencing was carried out according to the manufacturer’s protocol, using the EXP‐NBD104 together with the SQK‐LSK109 with a FLO‐FLG001 flow cell on a MinION sequencer (ONT, Oxford, UK).

Base calling was carried out with guppy (6.4.2). The resulting FASTQ files were aligned against a GRCh38 reference genome using minimap2 (2.24) and converted, sorted, and indexed with SAMtools (1.16). IGV (2.15.2) was used for data visualization. Variant calling was done with Clair3 (v0.1‐r12).

## Conflicts of Interest

Sebastian Kummer has received research support funding from Zealand Pharma and is involved in contract research with Rezolute Pharma (both manufacturing investigational products for use in congenital hyperinsulinism). Florian Kraft and Ingo Kurth received funding for sequencing by Oxford Nanopore Technologies (ONT) within the lonGER consortium (https://longer-consortium.de/) and the ELRIN network (https://www.elrin-network.eu/). Florian Kraft received travel reimbursement from Oxford Nanopore Technologies (ONT). The other authors declare no conflicts of interest.

## Author Contributions

Matthias Begemann: analyzed patient data, designed the experiments, and wrote the manuscript. Johannes Alexander Tobias Boy: revision of the manuscript. Florian Kraft: designed and analyzed LongRead experiments and data and wrote and revised the manuscript. Hendrik Vossschulte: provision of clinical data and revision of the manuscript. Winfried Barthlen: provision of clinical data and revision of the manuscript. Sebastian Kummer: detailed revision of the manuscript and clinical data. Thorsten Orlikowsky: provision of clinical data and revision of the manuscript. Adrin Torosoglu: provision of clinical data and intense revision of the manuscript. Thomas Eggermann: revision of the manuscript and analysis of MLPA data. Ingo Kurth: provision of clinical data and revision of the manuscript. Miriam Elbracht: provision of clinical data and revision of the manuscript. Angeliki Pappa: conception of the manuscript, clinical data, and revision of the manuscript.

## Funding

This research received no external funding.

## Data Availability

The datasets generated and/or analyzed during the current study are available from the corresponding author on reasonable request.
